# General practitioners' perceptions of the effectiveness of medical interventions: an exploration of underlying constructs

**DOI:** 10.1186/1748-5908-5-17

**Published:** 2010-02-16

**Authors:** Florian Vogt, David Armstrong, Theresa M Marteau

**Affiliations:** 1 Health Psychology Section, Department of Psychology, Institute of Psychiatry, King's College London, Bermondsey Wing, 5th Floor, London SE1 9RT, UK; 2 Department of General Practice, King's College London School of Medicine, King's College London, 5 Lambeth Walk, London SE11 6SP, UK

## Abstract

**Background:**

Many interventions shown to be effective through clinical trials are not readily implemented in clinical practice. Unfortunately, little is known regarding how clinicians construct their perceptions of the effectiveness of medical interventions. This study aims to explore general practitioners' perceptions of the nature of 'effectiveness'.

**Methods:**

The design was qualitative in nature using the repertory grid technique to elicit the constructs underlying the perceived effectiveness of a range of medical interventions. Eight medical interventions were used as stimuli (diclophenac to reduce acute pain, cognitive behaviour therapy to treat depression, weight loss surgery to achieve weight loss, diet and exercise to prevent type 2 diabetes, statins to prevent heart disease, stopping smoking to prevent heart disease, nicotine replacement therapy to stop smoking, and stop smoking groups to stop smoking). The setting involved face-to-face interviews followed by questionnaires in London Primary Care Trusts. Participants included a random sample of 13 general practitioners.

**Results:**

Analysis of the ratings showed that the constructs clustered around two dimensions: low patient effort versus high patient effort (dimension one), and small impact versus large impact (dimension two). Dimension one represented constructs such as 'success requires little motivation', 'not a lifestyle intervention', and 'health-care professional led intervention'. Dimension two represented constructs such as 'weak and/or minimal evidence of effectiveness', 'small treatment effect for users', 'a small proportion of users will benefit' and 'not cost-effective'. Constructs within each dimension were closely related.

**Conclusions:**

General practitioners judged the effectiveness of medical interventions by considering two broad dimensions: the extent to which interventions involve patient effort, and the size of their impact. The latter is informed by trial evidence, but the patient effort required to achieve effectiveness seems to be based on clinical judgement. Some of the failure of evidence-based medicine to be implemented may be more explicable if both dimensions were attended to.

## Background

Despite the overall success of evidence-based medicine in building a sound research underpinning for understanding the effectiveness of many medical interventions, its major failing has been getting those findings into clinical practice [1,2]. Interventions to increase implementation of evidence-based guidelines, using a wide-variety of methods including incentives, prompts, and education have had mixed results [3]. Critiques of this large literature highlight that many interventions lack explicit rationales or theoretical bases and insufficient piloting [2].

A wide variety of perceived barriers towards performing a clinical behaviour have been reported [4]. Among others, the perceived effectiveness of an intervention at achieving desired patient outcomes is considered an important determinant of behaviour [5-7]. A common response to this problem has been to increase clinicians' knowledge of an intervention's effectiveness (as derived from trial evidence) on the grounds that a rational clinician would want to provide the best treatment for his or her patients [5,6]. While the importance of the perceived effectiveness of medical interventions is well documented, little is known about the basis for these perceptions. Indeed, it is often assumed that perceived effectiveness simply reflects the clinician's understanding of the research evidence, in which case the problem lies in a failure to communicate the evidence in a way that makes sense. This may suggest efforts should be increased to communicate information about the effectiveness of an intervention in a more comprehensible manner to bridge such a communication gap. For example, information about an interventions' benefit is perceived differently depending on whether it is represented in relative or absolute terms [7].

An alternative explanation is that clinicians and researchers may not share the same meanings of the notion of effectiveness with clinicians considering factors that are not part of the formal evidence base of effectiveness. Research aimed at identifying the reasons behind suboptimal implementation may therefore consider in more detail how clinicians derive their perceptions of the effectiveness of medical interventions. In short, the communication gap identified in implementation studies may reflect different underlying constructs of effectiveness; that is, the problem is a conceptual gap rather than one of communication and understanding.

A variety of methods for ascertaining perceptions or constructs exist, including repertory grid and focus group techniques, in-depth interviews, and survey questionnaires. The repertory grid technique allows individuals to determine their own personal range of descriptions relevant to the issue without imposing experimenter-determined constructs on the data set [8-10]. Exploring how a group of clinicians conceptualise the idea of effectiveness as applied to medical interventions using this technique is one way of understanding whether a communication or a conceptual gap underpins the implementation problem.

## Methods

### Design

An exploratory study was carried out using repertory grid, data reduction, and clustering techniques to elicit and categorise general practitioners' perceptions of the effectiveness of a range of medical interventions.

### Participants

The sample comprised general practitioners (GPs) working in southeast London. Invitation letters were sent to 200 randomly selected GPs that were registered in three local Primary Care Trusts (the list was obtained from the Primary Care Trusts) explaining the nature of the study and asking for willingness to be interviewed and complete a questionnaire. The inclusion criterion was being a registered GP; there were no exclusion criteria. Fifteen GPs replied to the invitation letters, and interviews were held with 13 (nine were male and four female). Two GPs were not interviewed because a suitable interview date and time could not be arranged. Following the interview, all 13 were sent a questionnaire via email or post, according to his or her preference, which 12 completed (eight were male and four female). One GP did not complete the questionnaire because of time constraints. Participants were reimbursed for their time with a €40 ($80, €50) book token. Data was collected from GPs between September 2007 and February 2008.

### Procedure

Eight interventions targeting a variety of medical conditions were selected to be used as stimuli to be shown to GPs (Table 1). The interventions were chosen in consultation with two GPs to represent a wide range of interventions and conditions, all of which had evidence of effectiveness. The names of the eight interventions were printed on separate laminated cards (20 cm by 10 cm).

**Table 1:** Eight interventions used as stimuli

1.	Statins to prevent heart disease [27].
2.	Diclophenac to reduce acute pain [28].
3.	Cognitive behaviour therapy (CBT) to treat depression [29].
4.	Stop smoking groups to stop smoking [20].
5.	Weight loss surgery to achieve weight loss [30].
6.	Stopping smoking to prevent heart disease [31].
7.	Diet and exercise to prevent type 2 diabetes [32].
8.	Nicotine replacement therapy (NRT) to stop smoking [21].

Interviews were conducted at the participants' place of work and audio-taped. These lasted between 20 and 40 minutes. Each participant was shown the intervention cards in triads, selected at random from the eight intervention cards. Participants were asked to identify two interventions in each triad that were similar with regards to their effectiveness and to describe what made them similar (similarity pole). After this, they were asked to describe what made the third intervention different (difference pole). For example, when presented with cards showing statins, stop smoking groups, and nicotine replacement therapy (NRT), a respondent might group the two stop smoking cards together because both involved a behaviour, thus 'strong cooperation from patient needed' (the similarity pole), and the third as not requiring a behaviour, thus 'strong cooperation not needed' (the difference pole). These two descriptions represented one bipolar personal construct of the effectiveness of medical interventions; in this example, whether cooperation was needed. When participants could elicit no further personal constructs in a triad, another triad was presented to them, again selected at random from the eight intervention cards. This process was continued until participants could elicit no further constructs.

The personal constructs elicited from all the participants were then reduced in number by using an inductive content analysis [11]. Six researchers were asked to group independently the personal constructs elicited from the GPs into more general constructs depending on their similarity; the number of general constructs the researchers could create was not restricted. A hierarchical cluster analysis combined the groupings from the six researchers using Ward's method and Euclidean distance within SPSS 15.0. The dendrogram and the agglomeration schedule, two key measures for assessing cluster analysis [12], were used to identify clusters of similar constructs. The six researchers then discussed and agreed labels for each of the clusters of constructs to reflect the underlying theme.

Interviewed GPs were then sent a questionnaire. The questionnaire asked the GP to rate the study's eight interventions on the clusters of constructs derived from the cluster analysis using seven-point scales (Additional file 1).

Ratings were analysed and mapped by generalised procrustes analysis (GPA). This technique is a form of principal components analysis, which assesses the variability in the data by identifying patterns that explain the most variance, thereby highlighting patterns or dimensions among participants' responses. GPA, unlike principal components analysis, maps individual level data, and permits the production of maps showing areas of consensus between individuals, and links between variables. For clarity of interpretation only the consensus maps are shown in the results section.

## Results

### Dimension creation

In total, 108 personal constructs were elicited by the GPs, ranging from three to 10 elicited personal constructs (median = 9); examples include: use a psychological approach to achieve outcome, success is highly dependent on patient motivation, intervention has impact on ailment, effectiveness has good value for money, existing statistical evidence of effectiveness, cause ill effects in more than 30% of subjects, strong cooperation from patient needed. In the subsequent content analysis, the six researchers formed groups from the personal constructs; groups ranged from 12 to 17 constructs in size (median = 15.5). A cluster analysis of these groupings showed that these were best represented in 11 clusters. Table 2 shows the 11 clusters with their labels.

**Table 2:** Eleven clusters of constructs identified in the cluster analysis

1.	'This intervention has robust evidence of effectiveness.' versus 'This intervention has **weak **and/or minimal **evidence of effectiveness.'**
2.	'This intervention has a large treatment effect for users.' versus 'This intervention has a **small treatment effect **for users.'
3.	'A large proportion of users will benefit from this intervention.' versus 'A **small proportion of users **will **benefit **from this intervention.'
4.	'Success requires a lot of motivation from the patient.' versus '**Success requires little motivation **from the patient.'
5.	'This is a biomedical intervention (treatment using drugs, radiation, or surgery).' versus 'This is **not a biomedical intervention.'**
6.	'This intervention is appealing to patients.' versus 'This intervention is **not appealing to patients.'**
7.	'The impact of this intervention can be precisely measured.' versus 'The impact of this intervention is **difficult to measure.'**
8.	'This is a lifestyle intervention (*e.g*., Diet and exercise education).' versus 'This is **not a lifestyle intervention.'**
9.	'This intervention is cost-effective.' versus 'This intervention is **not cost-effective.'**
10.	'This is a patient led intervention.' versus 'This is a **healthcare professional-led **intervention.'
11.	'This intervention brings long-term benefits.' versus 'This intervention only **helps in the short-term.'**

Note: Text highlighted in bold is used in the text and figures to describe the construct.

GPA revealed that the clusters were resolved in three dimensions with eigenvalues greater than one. GPA can give results that suggest a consensus between participants when there is none requiring a comparative test with random data that reflects the distributional structure of the data. The clusters were significantly different from chance as determined by the permutation test (p < 0.05), indicating that a true consensus space was achieved for each of these. Dimensions one (76%) and two (10%) accounted for the majority of the variance in the model, together explaining 86% of the variance. Dimension three was difficult to interpret because no construct loaded exclusively on this dimension. The final analyses therefore focused on the solution in two dimensions.

**Figure 1:**
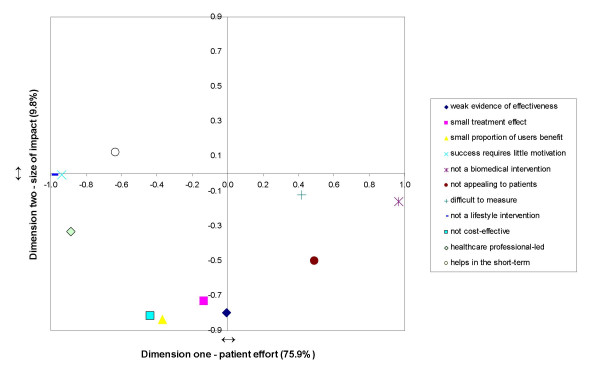
**Representation of constructs on dimensions 1 and 2**.

The two dimensions are shown in Figure 1 as orthogonal lines. Higher values (positive or negative) represent a stronger association of the cluster with the dimension. A number of clusters showed high or low scores on dimension one. These were whether the intervention was biomedical or not, whether it required patient motivation, whether it was a lifestyle intervention and whether the intervention was healthcare professional-led. All of these reflected whether or not the patient was involved in the treatment, and so dimension one was labelled as 'patient effort'. Dimension two grouped evidence of effectiveness, treatment effect, the proportion of users who might benefit, and cost effectiveness. This dimension was therefore labelled 'size of impact' of the intervention. Using these dimension labels, for example, the appearance of the construct cluster of whether the intervention appealed to patients in the bottom-right quadrant of the map suggests that GPs perceived such interventions as characterised by high patient effort and small impact.

**Figure 2:**
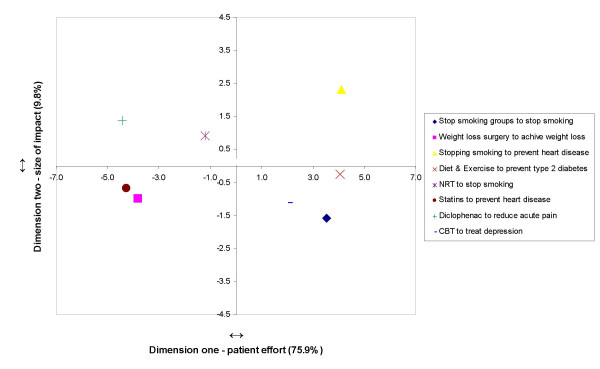
**Representation of interventions on dimensions 1 and 2**.

The eight interventions were also mapped along the two dimensions based on GPs' questionnaire-elicited ratings; the results are presented in Figure 2. Higher values (positive or negative) represent a stronger association of the intervention with the dimension. The distance between interventions reflects their degree of similarity with respect to the dimensions: the smaller the distance, the more similar the interventions are to each other. The top-right quadrant of the map reflecting high patient effort and large impact contained stopping smoking. The consensus representations of stop smoking groups, CBT, and diet and exercise fell within the bottom-right quadrant, reflecting high patient effort and small impact. The bottom-left quadrant of the map also reflects small impact low but in combination with low patient effort. The interventions statins and weight loss surgery appear in this quadrant. The top-left quadrant of the map reflects low patient effort and large impact and includes diclophenac and NRT. Assessment of residuals values for interventions, a measure of the disagreement between GPs about the individual interventions, showed little disagreement for most interventions (range: 89.0 and 135.8) apart from weight loss surgery (residual value = 226.8). Assessment of the individual plots (plots not shown) showed that while for some GPs weight loss surgery fell into the bottom-left quadrant, for others it was represented in the top-left quadrant; results suggesting a low consensus about the magnitude of the impact of weight loss surgery.

## Discussion

This paper explored a sample of GPs' views of the notion of effectiveness, and found this was underpinned by two dimensions, the 'size of impact' and 'patient effort needed'. The two dimensions appeared independent of each other. Semantically, the dimension 'size of impact' appears closely related to the estimates of effect size reported by trials, and is captured by constructs such as 'small treatment effect for users', 'a small proportion of users will benefit', or 'weak/minimal evidence of effectiveness'. The limited differentiation between these three constructs, as identified by the small separation on the maps, may be seen as reflecting a communication gap and the continued need for further education in medical statistics [13]. GPs in this study, however, also used another, independent dimension in judging an intervention's effectiveness, 'patient effort needed'. 'Patient effort needed' relates to the motivation and ability of the individual patient to achieve the desired outcome. The study thus provides evidence consistent with the hypothesis that a conceptual gap is a potential contributor to suboptimal implementation of evidence-based medicine.

Evidence based medicine involves integrating individual clinical expertise withthe best available external clinical evidence from systematicresearch; the gold standard for this evidence is the randomised clinical trial when it comes to answering whether an intervention is effective or not [14]. Trials, however, suffer from two major limitations in terms of their ready translation into clinical practice. The first is the role of patient behaviour and its generalisability beyond the trials. Early trials simply assessed the relative effectiveness of a particular intervention for those who received it. But although an intervention might be effective *in vitro*, so to speak, it may not be effective in the real world of clinical practice. This led to an emphasis on intention-to-treat designs that tried to capture the overall value of an intervention, recognising that some patients will not adhere to the intervention [15]. The influence of patient behaviour, however, in the form of involvement and motivation also precedes trials as the evaluation of effectiveness is only based on those patients who are recruited. Many patients are excluded (using formal criteria such as co-morbidities), and many more may decline to take part for a number of reasons, including the extra effort required, preferences for one arm of the trial, and refusal to be randomised [15]. The second major limitation of trials is the translation problem in moving between trial evidence, which is based on probabilities derived from populations, and the judgement about what is best for the individual patient [16].

Despite attempts to capture the influence of patient behaviour in trial design (such as in preference trials) and subgroup analyses (such as of adherence patterns), the randomisation underlying trials loses an important aspect of patient behaviour, and effectiveness is reported on average; yet there is considerable variability between patients' outcomes depending on their motivation and ability to adhere [17,18]. Findings of this study suggest that clinicians' appear to consider this. The degree of dependency on patient effort was also perceived as greater or smaller depending on the particular intervention. In other words, the confidence intervals around outcomes are perceived as wider for interventions that are more dependent on behaviour. A perception for which there may be supportive evidence: the impact of adherence on outcome is greater for non-medication regimes [19].

GPs may also perceive that the effectiveness of interventions obtained from clinical trials (comprising highly motivated participants) is attenuated in clinical practice (featuring moderately motivated patients), especially so, for interventions dependent on higher patient effort. The current results show, however, no clear evidence that interventions requiring high patient effort were systematically perceived as offering a smaller impact, even though stopping smoking was the only intervention perceived to involve high patient effort together with a large impact. An association between the two dimensions may have been confounded in the current study by using interventions with different levels of effectiveness. Future studies could present GPs with interventions of equal effectiveness and assess whether those perceived as requiring low patient effort are also characterised by a large impact.

The evidence on smoking cessation clinics, which have been shown to be 'effective', can illustrate the tension of patient effort needed and trial evidence. While trials have shown these clinics to be 'effective', trial patients are, in effect, screened for their motivation to quit and their willingness to make the extra effort required if they are to be recruited [20]. Six-month quit rates of control groups from trial of stop smoking groups who receive no intervention are often higher than would be expected from untreated quitters outside of the trials illustrating the likelihood of increased commitment and motivation [20]. Of those who do attend, commitment and motivation are clearly important for success, but the trial also reports the average effectiveness. Faced with this evidence, the clinician may consider whether his or her patient will attend a smoking clinic if referred, and whether they would regularly attend the support meetings. Deciding whether the patient is able and motivated to attend and adhere is therefore an important aspect of translating trial evidence into practice. Indeed, guidelines suggest that GPs only prescribe NRT to those they judge as motivated to make a quit attempt [21]. Even so, GPs may misjudge a patient's motivation when considering interventions such as the stop smoking groups.

In summary, randomised controlled trials tend to lose aspects of patient behaviour through the randomisation process. This means that adherence and other psychological influences on outcome are lost from the effectiveness equation, even though it has been shown in other studies to have a powerful therapeutic effect [22,23]. An intention-to-treat analysis can capture some of the patient effect, but trial inclusion still ignores the recruitment effect of trial participants. While evidence-based medicine is regarded as the integration of individual clinical expertise with external clinical evidence, this study suggests that GPs may consider that the external clinical evidence is not the same for all interventions, namely some evidence needs to be scrutinised with regards to patient effort more than others (*i.e*., some have a greater variability of outcome because of the patient effort required, and their replication may be less certain because the optimal trial conditions have a bigger impact on the overall outcome). In effect, clinicians in this study appear to be reassessing the formal effect size reported by trials in the light of perceived patient attributes. The implementation gap may be partly understandable when these limitations of trials are coupled with the tension of treating individual patients with interventions whose effectiveness is based on probabilities derived from the treatment of a population. However, whether clinicians' estimates of patients' effort required for the different interventions are accurate is not clear, and merits further study. The key role of assessments of 'patient effort needed' might also inform future research on the dissemination of trial evidence into everyday practice.

There are now a number of different models of how clinicians make decisions in everyday practice [24]. In each model, there are a series of 'inputs' in terms of existing knowledge and the new problem the patient presents that inform the decision-making process. Over the last two decades or so, evidence-based medicine has attempted to provide a key input into the process, usually through clinical guidelines, and there have been studies of the characteristics of guidelines that lead to greater impact [25]. A common assumption in this literature is that the idea of 'effectiveness' primarily derives from research evidence, and while it may be resisted for all sorts of local factors, the idea of what is effective remains largely unchallenged. What this paper has attempted to explore is whether the concept of 'effectiveness' has a common meaning. The results suggest that for these frontline clinicians the effectiveness embodied in research evidence needs to be filtered through an appreciation of patient characteristics that may increase or decrease the formal effect size of the intervention. An earlier qualitative study identified the importance of the patients' psychological and social needs in determining clinical decisions [26], and this new study using both qualitative and quantitative methods extends these findings and begins to pinpoint the key factor in the willingness and ability of patients to avail themselves of the intervention on offer.

To our knowledge this is the first study to examine what underlies GPs' perceived effectiveness of medical interventions. It is also novel in its use of qualitative participant-directed techniques supported by quantitative techniques to provide clear interpretations. Participants mentioned a wide range of constructs that they perceived as relevant without the researcher influencing either the choice of constructs or the choice of words to describe them. The repertory grid technique proved a useful framework for studying the perceived effectiveness of medical interventions, generating findings with face-validity. The study had several limitations. An alternative label could have been chosen for dimension one, to reflect more strongly the distinguishing feature of biomedical intervention versus non-biomedical interventions. The current label was chosen, first, because the constructs achieved near identical scores, and second, because it is the more inconclusive of the two, given that some interventions may be neither biomedical nor lifestyle interventions. The small sample size of qualitative studies limits their generalisability. Nevertheless, this study can be seen as an exploratory and innovative step in the understanding of how GPs construct the effectiveness of medical interventions, which may help to explain and address the gap in the implementation of research-based measures of effectiveness.

## Summary

This paper suggests that GPs hold a view of effectiveness that not only incorporates the dimension of clinical impact or effect size, which is provided by trial evidence, but also a 'patient effort needed' dimension, assessed on an individual patient basis. Some of the failure of evidence-based medicine to be implemented may be more explicable if both knowledge of effect size and estimates of patient effort needed were both seen to be part of the problem.

## Competing interests

The authors declare that they have no competing interests.

## Authors' contributions

FV conceived of the study, participated in its design and coordination, conducted the interviews, performed the analysis and interpretation, and drafted the manuscript. DA participated in its design, participated in the interpretation and redrafted the manuscript. TMM conceived of the study, participated in its design, participated in the interpretation, and helped draft the manuscript. All authors read and approved the final manuscript.

## Supplementary Material

Additional file 1 **Questionnaire**. Questionnaire to assess GPs' ratings of the study's eight interventions on the clusters of constructs derived from the cluster analysis. Click here for file
