# Supplementary material for: General practitioners' perceptions of the effectiveness of medical interventions: an exploration of underlying constructs
Source: Implement Sci. 2010 Feb 16;5:17. doi: 10.1186/1748-5908-5-17 (PMC2827366; doi:10.1186/1748-5908-5-17)
Supplement: Additional file 1 — Questionnaire. Questionnaire to assess GPs' ratings of the study's eight interventions on the clusters of constructs derived from the cluster analysis. [file 1748-5908-5-17-S1.PDF]

GP' attitudes towards the effectiveness of medical interventions

Please rate 8 medical interventions using the 11 differences mentioned most often in the interviews. In the layout you are asked to rate each medical intervention - one at a time. The layout is quick and easy to complete. Tick one box per row.

EXAMPLE:

If you **totally agree** that there is **"robust evidence"** for statins to prevent heart disease, just make a tick at 1.

Using statins to prevent heart disease. Please tick one box in each row.

	1	2	3	4	5	6	7	
this intervention has **robust evidence of effectiveness**	x							this intervention has **weak and/or minimal evidence of effectiveness**
this intervention has a **large treatment effect for users**						x		this intervention has a **small treatment effect for users**

If you **somewhat agree** that there is a **"small treatment effect for users"** from statins to prevent heart disease, just make a tick at 6.

1. Using statins to prevent heart disease. Please tick one box in each row.

	1	2	3	4	5	6	7	
this intervention has **robust evidence of effectiveness**								this intervention has **weak and/or minimal evidence of effectiveness**
this intervention has a **large treatment effect for users**								this intervention has a **small treatment effect for users**
**a large proportion of users will benefit** from this intervention								**a small proportion of users will benefit** from this intervention
**success requires a lot of motivation** from the patient								**success requires little motivation** from the patient
this is **a biomedical intervention** (treatment using drugs, radiation, or surgery)								this is **not a biomedical intervention**
this intervention is **appealing to patients**								this intervention is **not appealing to patients**
the impact of this intervention **can be precisely measured**								the impact of this intervention is **difficult to measure**
this is a **lifestyle intervention** (e.g. diet and exercise education)								this is **not a lifestyle intervention**
this intervention is **cost-effective**								this intervention is **not cost-effective**
this is a **patient led intervention**								this is a **health-care-professional led intervention**
this intervention brings **long-term benefits**								this intervention only **helps in the short-term**
	1	2	3	4	5	6	7	

2. Using Diclofenac 50mg to reduce acute pain. Please tick one box in each row.

	1	2	3	4	5	6	7	
this intervention has **robust evidence of effectiveness**								this intervention has **weak and/or minimal evidence of effectiveness**
this intervention has a **large treatment effect for users**								this intervention has a **small treatment effect for users**
**a large proportion of users will benefit** from this intervention								**a small proportion of users will benefit** from this intervention
**success requires a lot of motivation** from the patient								**success requires little motivation** from the patient
this is **a biomedical intervention** (treatment using drugs, radiation, or surgery)								this is **not a biomedical intervention**
this intervention is **appealing to patients**								this intervention is **not appealing to patients**
the impact of this intervention **can be precisely measured**								the impact of this intervention is **difficult to measure**
this is a **lifestyle intervention** (e.g. diet and exercise education)								this is **not a lifestyle intervention**
this intervention is **cost-effective**								this intervention is **not cost-effective**
this is a **patient led intervention**								this is a **health-care-professional led intervention**
this intervention brings **long-term benefits**								this intervention only **helps in the short-term**
	1	2	3	4	5	6	7	

3. Using cognitive behaviour therapy to treat depression. Please tick one box in each row.

	1	2	3	4	5	6	7	
this intervention has **robust evidence of effectiveness**								this intervention has **weak and/or minimal evidence of effectiveness**
this intervention has a **large treatment effect for users**								this intervention has a **small treatment effect for users**
**a large proportion of users will benefit** from this intervention								**a small proportion of users will benefit** from this intervention
**success requires a lot of motivation** from the patient								**success requires little motivation** from the patient
this is **a biomedical intervention** (treatment using drugs, radiation, or surgery)								this is **not a biomedical intervention**
this intervention is **appealing to patients**								this intervention is **not appealing to patients**
the impact of this intervention **can be precisely measured**								the impact of this intervention is **difficult to measure**
this is a **lifestyle intervention** (e.g. diet and exercise education)								this is **not a lifestyle intervention**
this intervention is **cost-effective**								this intervention is **not cost-effective**
this is a **patient led intervention**								this is a **health-care-professional led intervention**
this intervention brings **long-term benefits**								this intervention only **helps in the short-term**
	1	2	3	4	5	6	7	

4. Using stop smoking groups to stop smoking. Please tick one box in each row.

	1	2	3	4	5	6	7	
this intervention has **robust evidence of effectiveness**								this intervention has **weak and/or minimal evidence of effectiveness**
this intervention has a **large treatment effect for users**								this intervention has a **small treatment effect for users**
**a large proportion of users will benefit** from this intervention								**a small proportion of users will benefit** from this intervention
**success requires a lot of motivation** from the patient								**success requires little motivation** from the patient
this is **a biomedical intervention** (treatment using drugs, radiation, or surgery)								this is **not a biomedical intervention**
this intervention is **appealing to patients**								this intervention is **not appealing to patients**
the impact of this intervention **can be precisely measured**								the impact of this intervention is **difficult to measure**
this is a **lifestyle intervention** (e.g. diet and exercise education)								this is **not a lifestyle intervention**
this intervention is **cost-effective**								this intervention is **not cost-effective**
this is a **patient led intervention**								this is a **health-care-professional led intervention**
this intervention brings **long-term benefits**								this intervention only **helps in the short-term**
	1	2	3	4	5	6	7	

5. Using stomach surgery to achieve weight loss in obese adults. Please tick one box in each row.

	1	2	3	4	5	6	7	
this intervention has **robust evidence of effectiveness**								this intervention has **weak and/or minimal evidence of effectiveness**
this intervention has a **large treatment effect for users**								this intervention has a **small treatment effect for users**
**a large proportion of users will benefit** from this intervention								**a small proportion of users will benefit** from this intervention
**success requires a lot of motivation** from the patient								**success requires little motivation** from the patient
this is **a biomedical intervention** (treatment using drugs, radiation, or surgery)								this is **not a biomedical intervention**
this intervention is **appealing to patients**								this intervention is **not appealing to patients**
the impact of this intervention **can be precisely measured**								the impact of this intervention is **difficult to measure**
this is a **lifestyle intervention** (e.g. diet and exercise education)								this is **not a lifestyle intervention**
this intervention is **cost-effective**								this intervention is **not cost-effective**
this is a **patient led intervention**								this is a **health-care-professional led intervention**
this intervention brings **long-term benefits**								this intervention only **helps in the short-term**
	1	2	3	4	5	6	7	

6. Stopping smoking to prevent heart disease. Please tick one box in each row.

	1	2	3	4	5	6	7	
this intervention has **robust evidence of effectiveness**								this intervention has **weak and/or minimal evidence of effectiveness**
this intervention has a **large treatment effect for users**								this intervention has a **small treatment effect for users**
**a large proportion of users will benefit** from this intervention								**a small proportion of users will benefit** from this intervention
**success requires a lot of motivation** from the patient								**success requires little motivation** from the patient
this is **a biomedical intervention** (treatment using drugs, radiation, or surgery)								this is **not a biomedical intervention**
this intervention is **appealing to patients**								this intervention is **not appealing to patients**
the impact of this intervention **can be precisely measured**								the impact of this intervention is **difficult to measure**
this is a **lifestyle intervention** (e.g. diet and exercise education)								this is **not a lifestyle intervention**
this intervention is **cost-effective**								this intervention is **not cost-effective**
this is a **patient led intervention**								this is a **health-care-professional led intervention**
this intervention brings **long-term benefits**								this intervention only **helps in the short-term**
	1	2	3	4	5	6	7	

7. Using a lifestyle education program (diet and exercise) to prevent type 2 diabetes. Please tick one box in each row.

	1	2	3	4	5	6	7	
this intervention has **robust evidence of effectiveness**								this intervention has **weak and/or minimal evidence of effectiveness**
this intervention has a **large treatment effect for users**								this intervention has a **small treatment effect for users**
**a large proportion of users will benefit** from this intervention								**a small proportion of users will benefit** from this intervention
**success requires a lot of motivation** from the patient								**success requires little motivation** from the patient
this is **a biomedical intervention** (treatment using drugs, radiation, or surgery)								this is **not a biomedical intervention**
this intervention is **appealing to patients**								this intervention is **not appealing to patients**
the impact of this intervention **can be precisely measured**								the impact of this intervention is **difficult to measure**
this is a **lifestyle intervention** (e.g. diet and exercise education)								this is **not a lifestyle intervention**
this intervention is **cost-effective**								this intervention is **not cost-effective**
this is a **patient led intervention**								this is a **health-care-professional led intervention**
this intervention brings **long-term benefits**								this intervention only **helps in the short-term**
	1	2	3	4	5	6	7	

8. Using nicotine replacement patches/gum to stop smoking. Please tick one box in each row.

	1	2	3	4	5	6	7	
this intervention has **robust evidence of effectiveness**								this intervention has **weak and/or minimal evidence of effectiveness**
this intervention has a **large treatment effect for users**								this intervention has a **small treatment effect for users**
**a large proportion of users will benefit** from this intervention								**a small proportion of users will benefit** from this intervention
**success requires a lot of motivation** from the patient								**success requires little motivation** from the patient
this is **a biomedical intervention** (treatment using drugs, radiation, or surgery)								this is **not a biomedical intervention**
this intervention is **appealing to patients**								this intervention is **not appealing to patients**
the impact of this intervention **can be precisely measured**								the impact of this intervention is **difficult to measure**
this is a **lifestyle intervention** (e.g. diet and exercise education)								this is **not a lifestyle intervention**
this intervention is **cost-effective**								this intervention is **not cost-effective**
this is a **patient led intervention**								this is a **health-care-professional led intervention**
this intervention brings **long-term benefits**								this intervention only **helps in the short-term**
	1	2	3	4	5	6	7	
